# Exploring the Role of CD74 and D-Dopachrome Tautomerase in COVID-19: Insights from Transcriptomic and Serum Analyses

**DOI:** 10.3390/jcm12155037

**Published:** 2023-07-31

**Authors:** Nikola Ralchev Ralchev, Silviya Lyubenova Bradyanova, Yana Valerieva Doneva, Nikolina Mihaylova, Elena Vikentieva Elefterova-Florova, Andrey Ivanov Tchorbanov, José Francisco Munoz-Valle, Maria Cristina Petralia, Paola Checconi, Ferdinando Nicoletti, Paolo Fagone

**Affiliations:** 1 Department of Immunology, Institute of Microbiology, Bulgarian Academy of Sciences, 1113 Sofia, Bulgaria; 2 Department of Clinical Laboratory and Immunology, Military Medical Academy, 1606 Sofia, Bulgaria; 3 University Center for Health Science, Department of Molecular Biology and Genomics, University of Guadalajara, Guadalajara 49000, Jalisco, Mexico; 4 Department of Clinical and Experimental Medicine, University of Messina, 98122 Messina, Italy; 5 Department of Human Sciences and Quality of Life Promotion, San Raffaele University, 20132 Rome, Italy; 6 Department of Biomedical and Biotechnological Sciences, University of Catania, 95123 Catania, Italy

**Keywords:** CD74, D-DT, COVID-19, cytokines

## Abstract

The COVID-19 pandemic has posed a significant threat to public health worldwide. While some patients experience only mild symptoms or no symptoms at all, others develop severe illness, which can lead to death. The host immune response is believed to play a crucial role in determining disease severity. In this study, we investigated the involvement of CD74 and D-DT in COVID-19 patients with different disease severities, by employing an in silico analysis of a publicly available transcriptomic dataset and by measuring their serum levels by ELISA. Our results showed a significant increase in MIF levels in PBMCs from COVID-19 patients, as well as a significant increase in the D-DT levels in PBMCs. However, we observed no modulation in the serum levels of D-DT. We also observed a concordant reduction in the serum levels and PBMCs expression levels of CD74. Furthermore, we found a negative correlation between CD74 serum levels and IL-13. In conclusion, our study sheds light on the involvement of CD74 and D-DT in COVID-19, with potential implications for disease severity and treatment. Further studies are needed to fully elucidate the mechanisms underlying these observations and to explore the potential therapeutic value of targeting CD74 and IL-13 in COVID-19.

## 1. Introduction

COVID-19 is the result of an infection caused by the SARS-CoV-2 virus and can manifest with a diverse range of symptoms. Most people experience mild-to-moderate symptoms, but some progress to severe or critical disease that requires hospitalization. In some cases, patients develop acute respiratory disease syndrome (ARDS) and require mechanical ventilation. COVID-19 has a higher case fatality rate compared to seasonal influenza, meaning that a greater proportion of infected individuals die from the disease. In addition, SARS-CoV-2 infection is associated to de novo development of neurological and neuropsychiatric syndromes as well as to a long, subchronic form of the disease, termed Neuro-COVID, that may exhibit different clinical conditions, such as loss of smell and taste, headache, and fatigue, but also more severe symptoms, including encephalitis, stroke, and polyneuropathy [1]. The elderly and those with underlying medical conditions, such as cardiovascular disease, diabetes, chronic lung disease, chronic kidney disease, obesity, hypertension, or cancer, are at higher risk of mortality compared to healthy young adults. Increasing evidence suggests that a common mechanism accounting for the multiplicity of symptoms during SARS-CoV-2 infection is represented by a dysregulated immunoinflammatory response, similar to what has been observed in other coronaviruses, such as SARS and MERS. Current research is investigating how the immune system responds to SARS-CoV-2 and how to optimize this response to clear the virus [2,3,4,5,6,7,8].

The macrophage migration inhibitory factor (MIF) is a proinflammatory cytokine that is involved in various biological processes, including innate and adaptive immune responses, cell proliferation, and apoptosis. Its homologue, D-dopachrome tautomerase (D-DT), is a structurally similar protein that shares 30% sequence identity with MIF. Like MIF, D-DT is involved in various biological processes, including inflammation, tumorigenesis, and cell survival. Both MIF and D-DT have been implicated in the pathogenesis of various diseases, including autoimmune diseases, cancer, and infectious diseases [9,10,11,12,13,14,15,16,17,18,19,20,21,22,23,24,25,26,27].

MIF and D-DT exert their biological effects by binding to their receptors, CD74, CXCR2, and CXCR4. CD74 (also known as HLA-DR antigens-associated invariant chain) is a transmembrane protein expressed by immune cells, including macrophages, B cells, and dendritic cells. CXCR2 and CXCR4 are G-protein-coupled receptors that are expressed on various cell types, including leukocytes and endothelial cells. CD74 acts as the primary receptor for MIF, whereas CXCR2 and CXCR4 act as secondary receptors. D-DT also binds to CD74 but with lower affinity than MIF [9,10,11,12,13,14,15,16,17,18,19,20,21,22].

The proinflammatory effects of MIF and D-DT make these cytokines potential important players in the cascade of immunoinflammatory events that may lead to the several complications during SARS-CoV-2 infection and propel studies aimed at determining its role both as a pathogenetic and diagnostic cytokine and to eventually design and develop tailored therapeutic approaches [28,29,30,31,32].

Several studies have investigated the correlation between the macrophage migration inhibitory factor (MIF) and COVID-19 disease severity and prognosis. Aksakal et al. examined 110 COVID-19 patients and found significantly higher MIF levels in patients with moderate and severe disease compared to the control group. Moreover, MIF levels were higher in severe patients than in moderate patients, suggesting MIF as a potential indicator of disease prognosis in early infection stages [28]. Another study on 36 mechanically ventilated COVID-19 patients revealed that elevated MIF levels were associated with organ dysfunction and lower survival rates [29]. Additionally, MIF, along with biomarkers such as D-dimer, troponin, ferritin, and lactate dehydrogenase, predicted ICU admission of COVID-19 patients [30]. Furthermore, patients with COVID-19 exhibited lower frequency of the high-expression MIF CATT7 allele compared to healthy controls, but inpatients had a higher frequency of this allele than outpatients. Inpatients also had higher serum MIF levels correlated with ferritin and CRP levels [31]. Lastly, both classical and non-classical monocytes from recovering COVID-19 patients showed higher MIF expression levels [31]. Overall, these studies suggest that MIF plays a crucial role in predicting disease severity and outcome in COVID-19 patients, aiding in early risk assessment and personalized treatment strategies. However, no studies have yet analyzed the role of the MIF homologue, D-dopachrome tautomerase (D-DT), and of its receptors in COVID-19. In the present work, we have characterized the levels of these molecules and correlated them with different clinical characteristics of COVID-19 patients.

## 2. Materials and Methods

### 2.1. Transcriptomic Study

The whole-genome transcriptomic profile of PBMCs from COVID-19 patients and healthy donors was obtained from the GSE152418 dataset [33], downloaded from the public databank Gene Expression Omnibus (GEO; https://www.ncbi.nlm.nih.gov/gds) (accessed on 11 April 2023). The dataset included the transcriptomic profile from 17 healthy donors, 4 COVID-19 patients with moderate disease, 12 COVID-19 patients with severe disease (4 in ICU), and 1 convalescent COVID-19 patient (not included in the present study). The dataset was generated using the Illumina NovaSeq 6000 and analyzed using the web-based utility GREIN (https://shiny.ilincs.org/grein, accessed on 11 April 2023) [34]. GREIN employs edgeR implementation of the negative binomial generalized linear model to identify genes that exhibit differential expression between sample groups. The data were normalized using edgeR’s implementation of the trimmed mean of M-values (TMM) method. CPM values were used for all analyses, and genes were initially filtered using a threshold of CPM  >  0 in m samples, where m is the smallest sample size among any of the groups.

### 2.2. Patients

#### 2.2.1. COVID-19 Patients and Healthy Donors

Patients diagnosed with COVID-19 (n = 60), with laboratory confirmed SARS-CoV2 infection by RT-PCR, who attended the COVID-19 units of the Military Medical Academy in Sofia, were included in this study (34 males and 26 females). The mean age was 61.38 ± 14.53 years (min 25–max 98), and 33 patients were over 60 years. The mean length of hospital stay was 14.19 ± 5.88 days, ranging from 6 to 30 days. The control samples were obtained from 20 age- and sex-matched healthy blood donors. The study was approved by the local institutional ethics committee and all subjects signed an informed consent.

#### 2.2.2. Clinical Characteristics

Demographic information, clinical characteristics, laboratory results, and radiological findings were collected on hospital admission within 24 h. Laboratory results included hematology tests—WBC (white blood cells), lymphocytes (Ly), platelets (PLT), basophils (Ba), eosinophils (Eo) count, hemoglobin (Hgb); biochemistry tests—alanine aminotransferase (ALAT), aspartate aminotransferase (ASAT), lactic dehydrogenase (LDH), urea, creatinine; acute phase protein—ferritin, C-reactive protein (CRP); coagulation tests—prothrombin time (PT), activated partial thromboplastin time (aPTT), thrombin time (TT), fibrinogen, and D-dimer. The degree of lung involvement was assessed on hospital admission by chest radiography and/or computed tomography (CT).

The severity of COVID-19 was categorized as non-severe (mild), moderate, and severe (including severe and critical). The non-severe disease was characterized by mild clinical symptoms (uncomplicated upper respiratory tract infection, headache, fever) and normal oxygen saturation ≥95%. The moderate disease was defined by lower respiratory tract symptoms (pneumonia with no signs of severe disease), fever ≥ 38 °C, and oxygen saturation ≤ 95% on room air. The severe disease was defined by the presence of dyspnea (respiratory frequency ≥ 30 rate per minute), oxygen saturation < 90% in resting state, arterial partial pressure of oxygen (PaO2)/oxygen concentration (FiO2) ≤ 300, and unilateral or bilateral lung infiltrates > 50%. Patients with acute life-threating organ dysfunction requiring mechanical ventilation and intensive care unit (ICU) monitoring and treatment were defined as critical COVID-19 patients.

Clinical data for each of the patients recruited in this study are outlined in Table 1, Table 2 and Table 3.

#### 2.2.3. Blood Samples

Venous blood was collected from COVID-19 patients and healthy individuals in sterile blood tubes, and serum samples of 5 mL were collected from each subject using serum separator tubes (Vacutainer BD-Plymouth.PL67BP.UK, 5 mL) and stored at −80 °C.

#### 2.2.4. Enzyme-Linked Immunosorbent Assay (ELISA) for Soluble Serum CD74 and D-DT

Serum levels of human D-DT and CD74 (HLA class II histocompatibility antigen gamma chain) were detected by ELISA Kit according to the manufacturer’s instructions (MyBioSource, San Diego, CA, USA). For D-DT, detection range was 0.312–20 ng/mL and sensitivity 0.12ng/mL. For CD74, detection range was 0.625–40 ng/mL and sensitivity 0.375 ng/mL.

#### 2.2.5. Flow Cytometry Analysis of Th1, Th2, and Th17 Cytokines

Serum levels of human Th1, Th2, and Th17 cytokines were detected by LEGENDplex™ Human Th1/Th2 and Th17 Panel multi-Analyte Flow Assay Kits according to the manufacturer’s instructions (BioLegend, San Diego, CA, USA) by a BD LSR II flow cytometer (BD Biosciences, Mountain View, CA, USA). Briefly, the LEGENDplex™ Human Th1/Th2 and Th17 Panel multi-Analyte Flow Assay Kits are bead-based immunoassays that utilize the sandwich immunoassay principle. Beads of different sizes and fluorescence intensities are conjugated with specific antibodies and act as capture beads for target analytes. After binding, a biotinylated detection antibody cocktail is added, forming capture bead-analyte-detection antibody sandwiches. Streptavidin-phycoerythrin is introduced to generate fluorescent signals proportional to analyte concentration. By using a flow cytometer, analyte-specific populations can be separated and quantified.

The LEGENDplex™ Human Th1/Th2 kit allows the measurement of IL-2, IL-4, IL-5, IL-13, IL-6, IL10, IFNgamma, and TNFalpha, while the LEGENDplex™ Human Th17 kit allows the quantification of IL-6, IL-10, IL-17A, IL-17F, IL-22, IFNgamma, and TNFalpha.

Cytokines overlapping in the multi-Analyte Flow Assay Kits were averaged for each patient. We calculated the sum of IL-2, IL-6, IFNgamma, and TNFalpha for Th1 and the sum of IL-4, IL-5, IL-10, and IL-13 for Th2; Th17 was defined as the sum of IL-17A, IL-17F, and IL-22.

### 2.3. Statistical Analysis

Values reported in the figures correspond to mean ± SD. Flow cytometry results were analyzed by FlowJo software. Data were subjected to a Kolmogorov–Smirnov test, D’Agostino and Person Omnibus test, and Shapiro–Wilk normality test. According to the results from the normality tests, the Kruskal–Wallis test followed by Dunn’s post hoc test were applied to assess the statistical significance for the differences among groups. Correlation was performed using the non-parametric Spearman’s test. A value of *p* < 0.05 was considered statistically significant for all analyses. Statistical analyses were performed using the GraphPad Prism 9 software (San Diego, CA, USA).

## 3. Results

### 3.1. Transcriptomic Study

**Figure 1:**
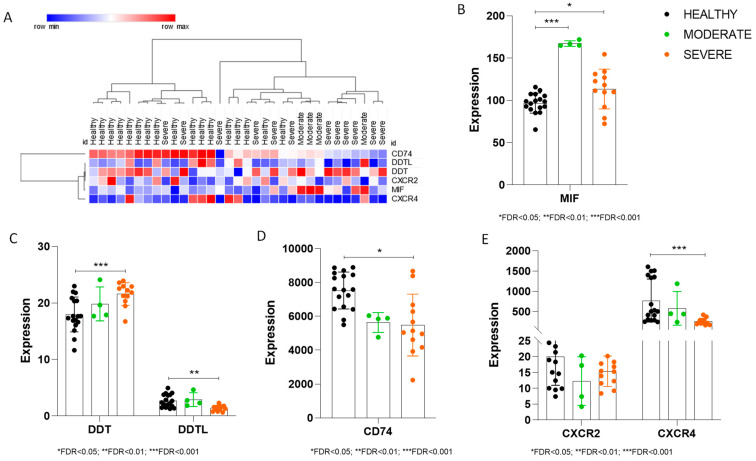
Transcriptomic analysis of PBMCs from COVID-19 patients, using the GSE152418 dataset. (**A**) Hierarchical clustering of the genes analyzed; (**B**) MIF levels in PBMCs from healthy donors and COVID-19 patients; (**C**) D-DT and D-DTL levels in PBMCs from healthy donors and COVID-19 patients; (**D**) CD74 levels in PBMCs from healthy donors and COVID-19 patients; (**E**) CXCR2 and CXCR4 levels in PBMCs from healthy donors and COVID-19 patients. Transcriptional levels are expressed in an arbitrary unit.

We made use of the whole-genome transcriptomic dataset GSE152418, generated on PBMCs from COVID-19 patients with different disease severity and healthy donors. We analyzed the expression levels of MIF, D-DT (D-dopachrome tautomerase), D-DTL (D-dopachrome tautomerase like), CD74, CXCR2, and CXCR4, as shown in Figure 1A.

Our analysis revealed a significant increase in MIF levels in both moderate and severe cases of COVID-19, as compared to PBMCs collected from healthy donors (Figure 1B). The highest increase in MIF expression was observed in PBMCs collected from patients with moderate disease (Figure 1B).

In a manner similar to MIF, the D-DT transcripts were also significantly augmented as compared to healthy controls in COVID-19 patients with moderate diseases, while only a trend toward upregulation was observed in patients with severe disease. Regarding D-DTL, the circulating levels were much lower than those of D-DT, in all investigated samples. A significant decrease was also observed in the PBMCs collected from patients with severe COVID-19, as compared to healthy samples (Figure 1C).

Similarly, CD74 was significantly lower in severe COVID-19 cases, while only a trend of reduction was found in the PBMCs collected from patients with moderate disease (Figure 1D). As regards the chemokine receptors, CXCR2 and CXCR4, we found significantly reduced levels of CXCR4 in severe COVID-19 patients and only a non-significant trend of reduction for CXCR2 (Figure 1E).

### 3.2. Patients

#### 3.2.1. COVID-19 Patients Distribution

The sera from 60 patients diagnosed with COVID-19 were separated into three groups (20 patients each) depending on the severity of disease: mild, moderate, and severe. The mild disease group included 14 men and 6 women. The mean age was 55.6 ± 15.6. The moderate disease group was composed of 10 men and 10 women, with a mean age of 62.7 ± 12.2. The severe COVID-19 group included 10 men and 10 women, with a mean age of 65.9 ± 14.7. Among them, 2/10 men and 6/10 women died of COVID-19.

#### 3.2.2. Serum CD74 and D-DT Levels in COVID-19 Patients

**Figure 2:**
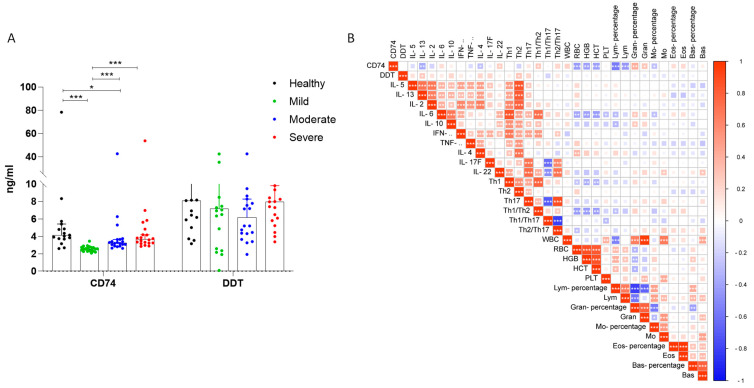
Evaluation of CD74 and D-DT in COVID-19 patients and healthy donors. (**A**) Levels of serum CD74 and D-DT in COVID-19 patients and healthy donors; (**B**) correlation between CD74, D-DT, serum cytokine levels and blood counts. * *p* < 0.05; ** *p* < 0.01; *** *p* < 0.001 by Spearman’s test.

We investigated the levels of CD74 and D-DT in the sera of COVID-19 patients and healthy donors by ELISA (Figure 2). The highest levels of CD74 were detected in healthy individuals. Significantly lower levels of CD74 were observed in mild and moderate COVID-19 patients, as compared to healthy donors. No significant differences were observed for the severe cases of COVID-19 in comparison to healthy people (Figure 2A). No significant differences in the D-DT levels were observed between the groups (Figure 2A).

We next conducted a correlation analysis for CD74, D-DT, and other clinical parameters, including cytokines and blood cell counts. We found that there was no significant correlation between the D-DT levels and the other included parameters. However, we did observe a significant negative correlation between CD74 and IL-13 (r = −0.3638; *p* = 0.0043). Moreover, we observed an inverse correlation between CD74 and several blood cell counts, including red blood cell count (RBG), hematocrit (HCT), hemoglobin (HGB), lymphocytes (Lymp), and monocytes (Mono). In contrast, we found a positive correlation between CD74 and granulocytes.

#### 3.2.3. Analysis of CD74 and D-DT Levels in Recovered Cases and Lethal Cases from Severe COVID-19 Patients

**Figure 3:**
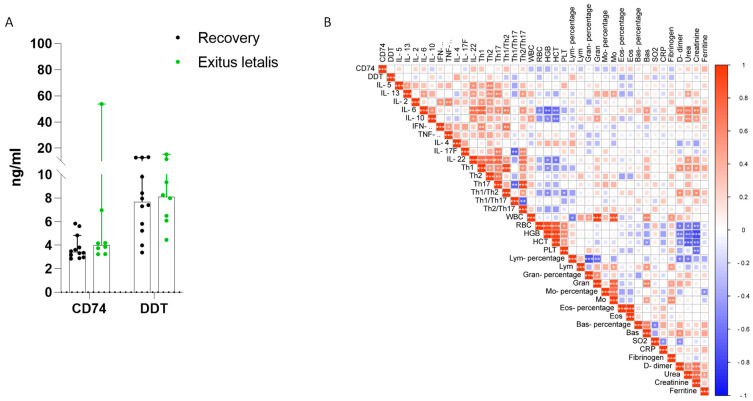
Evaluation of CD74 and D-DT in severe COVID-19 cases. (**A**) Levels of serum CD74 and D-DT in recovery and lethal cases of COVID-19; (**B**) correlation between CD74, D-DT, serum cytokine levels and blood counts. * *p* < 0.05; ** *p* < 0.01; *** *p* < 0.001 by Spearman’s test.

We next aimed to investigate whether there were differences in the serum levels of D-DT and CD74 between recovered cases and lethal cases of severe COVID-19 patients. The results showed no significant differences in the levels of D-DT or CD74 between the recovered and lethal cases of COVID-19 (Figure 3A).

Furthermore, we aimed to determine whether there were correlations between the levels of D-DT and CD74 and other investigated cytokines and blood cell counts in severe cases of COVID-19. The results showed no significant correlation between D-DT and any of the other investigated proteins or blood cell counts (Figure 3B).

## 4. Discussion

In the present study, we aimed to investigate the potential involvement of CD74 and D-DT and their receptors in COVID-19 patients with different disease severity, by employing an in silico analysis of a publicly available transcriptomic dataset and by measuring their serum levels by ELISA. Previous studies have already explored the role of MIF in COVID-19 [28,29,31,35], and we aimed at building upon those observations in order to provide a more comprehensive understanding of the role of the MIF pathway in COVID-19. Specifically, we sought to investigate the expression levels of CD74 and D-DT in COVID-19 patients and to examine their potential association with disease severity.

D-dopachrome tautomerase (D-DT), identified as a homolog of MIF, is located on chromosome 22q11.23. Although D-DT and MIF share only 34% sequence homology, they exhibit strong structural similarities. Notably, both proteins possess enzymatic binding pockets containing a catalytic proline residue, allowing them to convert substrates such as D-dopachrome and p-hydroxyphenylpyruvate (HPP) into different end products. D-DT produces 5,6-dihydroxyindole, while MIF generates 5,6-dihydroxyindole carboxylic acid. Additionally, D-DT binds to the CD74 ectodomain but with a higher dissociation constant and dissociation rate compared to MIF. It is interesting to note that D-DT lacks the motif necessary for MIF to bind to the chemokine receptor, CXCR2.

Despite their structural and biochemical similarities, the exact biological functions of MIF and D-DT are still not fully understood. However, there is growing evidence suggesting their synergistic effects in various biological contexts [9].

When mice were subjected to the LPS challenge, a notable increase in the D-DT levels in the serum was observed, peaking at 16 h. Interestingly, the kinetics of D-DT elevation in the serum mirrored those of MIF. Furthermore, both D-DT and MIF were detected in the serum at similar concentrations. At the baseline, the measured levels were approximately 6 ng/mL for D-DT and around 2 ng/mL for MIF. However, the levels peaked at approximately 30 ng/mL for the D-DT and approximately 40 ng/mL for MIF. These findings are significant because it is worth noting that in cultured macrophages, MIF is produced at levels 20 times higher than the D-DT. This suggests that in vivo, during systemic inflammation, cells other than macrophages serve as an important source of D-DT. These new insights indicate the different involvement of MIF and D-DT in the inflammatory response beyond its association with macrophages.

Taking into account the similarities and differences between MIF and D-DT, it becomes conceivable to explore tailored therapeutic approaches for COVID-19 that involve MIF-D-DT modulation. However, a comprehensive understanding of the protective or pathogenic roles of these cytokine homologs is paramount before implementing such strategies. Further research is necessary to fully unravel the biological functions of both MIF and D-DT, as well as their implications in COVID-19. Once their roles are clarified, targeted treatments can be developed to benefit specific subsets of COVID-19 patients.

Our analysis of the transcriptomic dataset GSE152418 showed a significant increase in MIF levels in PBMCs from COVID-19 patients with moderate and severe disease, as compared to healthy donors’ PBMCs. Specifically, the highest increase in MIF was observed in patients with moderate disease. These findings are consistent with previous studies reporting elevated MIF levels in COVID-19 [29,30,31,35] and suggest a potential role of MIF in the pathogenesis of COVID-19.

D-DT is a multifunctional protein involved in various biological processes, including immune response, inflammation, and oxidative stress. In our study, we observed a significant increase in the D-DT expression levels in PBMCs from COVID-19 patients compared to healthy donors. However, no modulation was observed in the serum levels of the protein. This discrepancy between the serum and the PBMCs levels of D-DT needs to be further investigated. Nonetheless, previous studies have reported that D-DT plays a role in the regulation of immune response and inflammation, which suggests its potential involvement in the pathogenesis of COVID-19.

The lack of significant modulation of D-DT serum levels in COVID-19 patients, despite its increased transcriptional levels in PBMCs, is an interesting observation. One possible explanation could be that D-DT has a different expression pattern in different cell types. While PBMCs show increased transcriptional levels of D-DT, other cell types, such as hepatocytes and adipocytes, may be responsible for the secretion of D-DT into the bloodstream. Another possibility could be that D-DT is rapidly cleared from the serum, leading to lower levels, despite increased production in PBMCs. Furthermore, D-DT has a short half-life in the blood, with a reported half-life of approximately 60 min [36]. This short half-life could contribute to the lack of significant modulation of D-DT serum levels in COVID-19 patients. Additionally, it is possible that D-DT is sequestered or metabolized in the lungs or other organs affected by COVID-19, leading to lower levels in the serum. Another potential explanation could be that the increased transcriptional levels of D-DT in PBMCs do not necessarily reflect increased protein production. Post-transcriptional mechanisms such as mRNA stability, translation efficiency, and protein turnover can all impact protein levels, and these factors may differ between PBMCs and other cellular populations [37]. Further studies are needed to fully understand the mechanisms underlying the observed differences in D-DT levels between PBMCs and serum in COVID-19 patients.

CD74, also known as the HLA-DR antigens-associated invariant chain, is a chaperone protein involved in the trafficking and presentation of major histocompatibility complex (MHC) class II molecules. Our study shows a concordant reduction in both serum levels and PBMCs expression levels of CD74 in COVID-19 patients, particularly those with severe disease. Furthermore, we observed a negative correlation between CD74 serum levels and IL-13, a cytokine that has been recently described to be pathogenetic in COVID-19 [38,39,40,41].

IL-13 is a pleiotropic cytokine produced by various immune cells, including Th2 cells, eosinophils, and mast cells. It exhibits functions similar to IL-4 and inhibits macrophages [42]. IL-13 has been implicated in the pathogenesis of various inflammatory and allergic diseases, including asthma, allergic rhinitis, and atopic dermatitis. Recently, studies have reported that elevated levels of IL-13 are associated with the severity of COVID-19. In addition, patients who developed COVID-19 while prescribed Dupilumab, a monoclonal antibody that blocks IL-4 and IL-13 signaling, have been shown to have a less severe disease course. Furthermore, in SARS-CoV-2-infected mice, IL-13 neutralization reduced death and disease severity, demonstrating an immunopathogenic role for this cytokine. Following anti-IL-13 treatment in infected mice, hyaluronan synthase 1 (Has1) was the most downregulated gene in the lung, and hyaluronan accumulation was decreased. These findings suggest that IL-13 promotes the accumulation of hyaluronan in the lung, contributing to the development of respiratory failure in COVID-19 [38,39,40,41]. This is, somehow, in contrast to the previously demonstrated protective role of exogenous IL-13 in rodent models of LPS-induced endotoxemia [42,43,44] that shares some similar immunopathogenetic mechanisms as SARS-CoV-2 pneumonitis. The reasons for the possible pathogenetic role of endogenous IL-13 in COVID-19 vs. LPS-induced lethality remains to be studied.

The negative correlation between CD74 serum levels and IL-13 found in our study suggests a potential role of CD74 in regulating the IL-13-mediated immune response and inflammation in COVID-19. CD74 has been shown to play a role in the regulation of MHC class II presentation and antigen processing, and it has been reported to modulate the immune response and inflammation in various diseases. These findings suggest that CD74 may be involved in the inflammatory response to COVID-19, although it may not be useful as a prognostic marker for severe COVID-19 disease, and that further studies are needed to explore the potential role of CD74 in the pathogenesis of COVID-19 and the feasibility of its use as a therapeutic target. It is also interesting to note that in the transcriptomic studies, the significantly reduced expression of the MIF co-receptor CXCR4 paralleled that of CD74, suggesting that a common mechanism of downregulated expression could occur during moderate and severe cases of COVID-19. The exact comprehension of the biological significance of downregulated expression secretion of MIF and D-DT receptors during COVID-19 infection remains to be established and requires careful consideration.

It will be worth investigating, in future studies, whether CD74 and D-DT are involved in the long-term immune response to SARS-CoV-2. While much of the focus in the early stages of the pandemic has been on the acute phase of COVID-19, there is growing recognition of long-term health consequences in survivors of the disease, often referred to as “Long COVID”. As discussed above, recent studies have indeed found that patients with Long COVID and Neuro-COVID in particular had a persistent immune response to SARS-CoV-2, with elevated levels of specific cytokines and chemokines [45]. It would be interesting to investigate whether MIF, D-DT, and their receptors are also involved in this persistent immune response, and whether they could be potential targets for therapies aimed at mitigating long-term-health COVID-19 consequences. In particular, the potential pathogenetic role of MIF and D-DT in Neuro-COVID is consistent with several data, generated by us and others, indicating an important role of these cytokines in neurological and psychiatric conditions characterized by sustained immune-inflammation, including Multiple Sclerosis [46,47,48,49] and Guillain Barré syndrome [50]. In this regard, Laudanski and colleagues have observed that MIF serum levels differentiated patients with cerebrovascular events from those who did not have a stroke during the acute phase of COVID-19 [51]. It is of interest in this context and as regards the translatability of the emerging finding of MIF and D-DT in COVID-19 that there are two clinical studies that aim at evaluating the effects of the semi-specific inhibitor of MIF, Ibudilast, on the course of the disease (NCT04429555; NCT05513560) and one specifically designed to treat patients with lingering symptoms of COVID-19 (Long COVID).

While this study hints for the first time at the involvement of CD74 and D-DT in COVID-19, there are some limitations that do not allow to establish more robust conclusions regarding the role of these factors in disease severity and treatment. To establish conclusive evidence and generalize the findings, it is crucial to conduct larger-scale studies with longer follow-up periods. These studies should include diverse populations to account for demographic and clinical variations. Moreover, replication of the findings in independent cohorts would enhance the reliability and validity of the results. In addition, a small study period may not capture the full spectrum of disease progression and variations in immune responses over time. COVID-19 is a dynamic disease with diverse outcomes, and studying patients over a longer period would provide a more comprehensive understanding of the involvement of CD74 and D-DT in different disease stages. Similarly, the relatively small number of cases studied herein may not provide a representative sample of the population, limiting the generalizability of the findings. With the present sample size, there is an increased risk of sampling bias, which can affect the reliability of the results. Ideally, studies should include larger cohorts to ensure statistical power and account for potential confounding factors.

Despite these limitations, the present study, for the first time, provides insight into the role of CD74 and D-DT in the immune response to SARS-CoV-2 infection and highlights the potential for these proteins to be targeted in the development of new therapies for COVID-19. Future research should continue to explore the complex interplay between these proteins and other cytokines and chemokines involved in the immune response to SARS-CoV-2, as well as their potential role in the long-term immune response to the virus.

## Figures and Tables

**Table 1:** (A) Clinical data for the COVID-19 patients with mild disease—data 1. (B) Clinical data for the COVID-19 patients with mild disease—data 2.

A.										
**ID Sample**	**Age**	**Gender**	**WBC**	**RBC**	**HGB g/L**	**HCT**	**PLT**	**Lym%**	**Lym#**	**Gran%**
2	51	woman	4.63	5.6	133	41.8	280	40.4	1.87	48.4
5	44	woman	10.04	4.49	135	39.7	241	28.3	2.84	63.1
6	45	man	8.61	5.66	159	46.7	265	27.1	2.33	63.3
8	49	man	9.73	5.08	152	44.8	289	28.7	2.79	61.7
12	72	man	6.13	5.18	169	48.1	106	18.6	1.14	77.3
14	52	man	9.01	5.4	163	48.2	376	9.2	0.83	75.8
15	61	man	5.24	5.11	161	45.9	219	27.9	1.46	63.5
40	40	man	8.13	5.3	148	44.3	261	9.1	0.74	86.5
46	39	man	2.55	4.74	139	40.9	185	24.7	0.63	67.5
48	75	man	14.98	3.52	108	33.3	262	13.8	2.07	81.4
67	57	man	5.68	4.86	146	42.3	91	31.3	1.78	56.5
75	47	woman	6.55	5.66	150	45.6	236	25.3	1.66	68.5
76	50	man	6.32	4.18	153	44.2	204	16.9	1.07	72.8
78	80	man	14.95	4.19	141	40.7	440	14.4	2.15	86.8
85	75	man	6.23	5.68	165	48.3	174	39.5	2.46	48.4
92	48	man	3.9	4.76	146	42.1	195	28.7	1.12	61.8
93	83	woman	3.94	4.51	140	4	138	32	1.26	59.8
94	70	man	10.68	5.07	135	41.6	219	5.3	0.57	90
96	48	woman	7.66	3.91	109	34.2	354	17.2	1.32	73.1
99	25	woman	5.24	5.05	144	44.5	173	16.6	0.87	80.3
B.										
**ID Sample**	**Age**	**Gran#**	**Mo%**	**Mo#**	**Eo%**	**Eo#**	**Ba%**	**Ba#**	**SO2%**	**Fever**
2	51	2.24	11	0.51	0	0	0.2	0.01	96%	38.6
5	44	6.34	8.2	0.82	0.2	0.02	0.2	0.02	98%	37.6
6	45	5.45	8.1	0.7	1.3	0.11	0.2	0.02	95%	37.3
8	49	6	8.4	0.82	0.9	0.09	0.9	0.09	98%	37.7
12	72	4.74	3.9	0.24	0	0	0.2	0.01	95%	37.4
14	52	6.83	14.9	1.34	0	0	0.1	0.01	96%	37.9
15	61	3.33	8.2	0.43	0.2	0.01	0.2	0.01	97%	37.8
40	40	7.03	4.4	0.36	0	0	0	0	97%	38
46	39	1.72	7.8	0.2	0	0	0	0	96%	37.7
48	75	12.19	4.1	0.62	0.4	0.06	0.3	0.04	97%	37.2
67	57	3.21	12	0.68	0	0	0.2	0.01	97%	38
75	47	4.49	6	0.39	0	0	0.2	0.01	96%	38.6
76	50	4.6	9.5	0.6	0.5	0.03	0.3	0.02	97%	37.3
78	80	12.97	8.1	1.21	0.3	0.05	0.4	0.06	98%	37.8
85	75	3.02	11.6	0.72	0	0	0.5	0.03	96%	37.8
92	48	1.12	9	0.35	0	0	0.5	0.02	96%	37.9
93	83	2.36	7.9	0.31	0	0	0.3	0.01	97%	37
94	70	9.61	4.6	0.49	0	0	0.1	0.01	96%	37.4
96	48	5.6	9.7	0.74	0	0	0	0	95%	37.6
99	25	4.21	2.9	0.15	0	0	0.2	0.01	98%	37.8

**Table 2:** (A) Clinical data for the COVID-19 patients with moderate disease—data 1. (B) Clinical data for the COVID-19 patients with moderate disease—data 2.

A.										
**ID Sample**	**Age**	**Gender**	**WBC**	**RBC**	**HGB g/L**	**HCT**	**PLT**	**Lym%**	**Lym#**	**Gran%**
1	58	woman	7.63	4.32	122	37.1	197	8	0.61	86.9
3	58	woman	10.28	3.97	119	34.6	295	10.1	1.04	86.3
4	77	man	8.23	2.83	95	28.3	170	1.8	0.15	94.2
11	58	woman	5.44	4.36	132	39.2	241	27.8	1.51	63.9
18	66	man	16.93	5.11	151	44.4	400	4.3	0.72	88.7
20	73	man	5.08	3.78	120	35.8	247	12	0.61	80.7
23	45	man	8.62	4.94	142	39.8	229	13.5	1.16	83
24	68	man	7.85	4.83	168	46.9	176	16.1	0.51	76.9
34	84	man	11.1	3.58	111	33.5	379	7.5	0.83	83.6
41	82	woman	11.32	3.34	99	31.4	210	9.2	1.04	84.6
43	42	woman	5.73	3.48	106	32.1	351	13.8	0.79	80.6
44	60	woman	6.59	4.9	136	40.1	328	20.6	1.36	76.5
45	44	man	7.92	4.38	137	38.4	220	16.5	1.31	80
47	67	woman	11.33	4.08	132	0.38	204	4.1	0.46	91.8
49	51	man	8.78	4.86	150	44.6	266	21.4	1.88	72.5
52	65	woman	5.97	4.07	120	35.8	364	22.9	1.37	66
56	76	woman	5.11	4.12	131	38.1	187	14.9	0.76	81.2
57	51	man	6.01	4.92	142	24.5	373	20.8	1.25	67.8
59	63	woman	5.89	3.92	124	35.8	161	17.3	0.33	76.4
60	66	man	22.06	4.86	139	40	416	4.1	0.9	92.2
B.										
**ID Sample**	**Age**	**Gran#**	**Mo%**	**Mo#**	**Eo%**	**Eo#**	**Ba%**	**Ba#**	**SO2%**	**Fever**
1	58	6.63	5.1	0.39	0	0	0	0	88%	39
3	58	8.87	3.5	0.36	0	0	0.1	0.01	90%	39.5
4	77	7.75	4	0.33	0	0	0	0	94%	39.2
11	58	3.48	7.9	0.43	0	0	0.4	0.02	92–93%	38.2
18	66	15.03	6.6	1.12	0	0	0.4	0.06	92%	38.4
20	73	4.1	7.1	0.36	0	0	0.2	0.01	91%	38.5
23	45	7.15	3.2	0.28	0	0	0.3	0.03	85%	38
24	68	6.04	6.5	0.51	0.1	0.01	0.4	0.03	90%	38.3
34	84	9.28	7.4	0.82	1.4	0.16	0.1	0.01	90%	39
41	82	9.58	6.2	0.7	0	0	0	0	93%	38.7
43	42	4.62	5.6	0.32	0	0	0	0	88–92%	39.4
44	60	5.04	2.7	0.18	0	0	0.2	0.01	90%	39
45	44	6.33	16.5	0.26	0.1	0.01	0.1	0.01	88–89%	37.9
47	67	10.41	4	0.45	0	0	0.1	0.01	93%	38.3
49	51	6.36	6	0.53	0	0	0.1	0.01	91%	39.4
52	65	3.93	9.5	0.57	1.3	0.08	0.3	0.02	93–94%	38.2
56	76	4.15	3.9	0.2	0	0	0	0	90%	39.5
57	51	4.07	10.3	0.62	0.08	0.05	0.3	0.02	94–95%	38
59	63	4.5	5.6	0.33	0.2	0.01	0.5	0.03	94%	38.6
60	66	20.34	3.5	0.77	0	0	0.2	0.05	92%	38.5

**Table 3:** (A) Clinical data for the COVID-19 patients with severe disease—data 1. (B) Clinical data for the COVID-19 patients with severe disease—data 2. (C) Clinical data for the COVID-19 patients with severe disease—data 3.

A.										
**ID Sample**	**Age**	**Gender**	**WBC**	**RBC**	**HGB g/L**	**HCT**	**PLT**	**Lym%**	**Lym#**	**Gran%**
7	64	man	9.69	4.57	125	38.1	209	12.1	1.17	71.2
9	53	man	6.65	3.55	120	35.5	85	6.8	0.45	90.7
22	77	man	2.6	3.76	118	38.8	26	6.5	0.17	59.6
26	50	man	2.67	4.54	138	42.9	161	3.2	0.94	58.1
27	74	woman	14.28	4.35	133	38.8	325	2.7	0.39	92.7
32	79	woman	32.19	1.29	40	11.4	96	2.5	0.79	93.9
36	48	man	7.42	4.84	147	44	330	10.1	0.74	86.7
37	75	woman	42.3	2.4	74	23.1	132	3.4	1.44	85.3
38	43	man	6.76	5.13	147	44.7	270	6.4	0.43	89.9
51	73	man	10.54	4.81	133	38.9	310	8.4	0.89	83.1
53	98	man	6.68	3.93	124	36.7	185	13	0.87	81.5
54	48	woman	6.79	4.61	136	40.3	227	16.5	1.12	75
63	79	woman	11.68	4.01	122	36.6	263	9.9	1.16	84.6
65	66	woman	10.5	4.37	128	38.6	320	7.3	0.77	89.5
66	72	woman	16.34	4.49	134	39.8	245	7.7	1.26	86.8
77	82	woman	16.17	4.67	127	38.2	272	3.6	0.58	89.8
80	49	man	10.6	5.21	152	45.2	226	8	0.85	86.8
84	64	woman	11.29	4.71	140	41.5	287	9.3	1.05	81.5
89	62	man	3.61	3.43	109	32	106	10.5	0.38	84
91	62	woman	9.7	4.39	123	35.7	233	3.7	0.36	89.9
B.										
**ID Sample**	**Age**	**Gran#**	**Mo%**	**Mo#**	**Eo%**	**Eo#**	**Ba%**	**Ba#**	**SO2%**	**Fever**
7	64	6.89	4.4	0.43	0.6	0.06	0.4	0.04	82%	39
9	53	6.04	2.3	0.15	0	0	0.2	0.01	86%	38.4
22	77	1.55	2.7	0.07	30.8	0.8	0.4	0.01	79%	39
26	50	1.55	6.7	0.18	0	0	0	0	86%	38
27	74	13.23	4.5	0.64	0	0	0.1	0.02	81%	39
32	79	30.25	3.3	0.73	1.2	0.38	0.1	0.04	87%	39
36	48	6.43	3.2	0.24	0	0	0.1	0.01	83%	39.5
37	75	36.08	11.1	4.69	0	0	0.2	0.09	82%	39.6
38	43	6.08	1.5	0.1	2.1	0.14	0.1	0.01	87%	39.4
51	73	8.75	8.3	0.88	0.1	0.01	0.1	0.01	88%	38.9
53	98	5.44	4.5	0.3	0.9	0.06	0.1	0.01	80%	38
54	48	5.09	8.2	0.56	0	0	0.3	0.02	82%	38.5
63	79	9.87	4.3	0.5	0.9	0.11	0.3	0.04	63%	37.5
65	66	9.39	3	0.32	0	0	0.2	0.02	65%	39.3
66	72	14.18	5.4	0.88	0	0	0.1	0.02	77%	37.9
77	82	14.53	6.4	1.03	0	0	0.2	0.03	80%	38
80	49	9.2	5.2	0.55	0	0	0	0	83%	40
84	64	9.21	7.2	0.81	1.9	0.21	0.1	0.01	86%	39
89	62	3.03	5.5	0.2	0	0	0	0	83%	37.4
91	62	8.71	6.3	0.61	0.1	0.01	0.1	0.01	87%	38
C.										
**ID Sample**	**Age**		**CRP**	**Fibrinogen**		**D-Dimer**		**Urea**	**Creatinine**	**Ferritin**
7	64	recovery	201.2	6.86		1.32		8.1	124	2042
9	53	recovery	5.4	4.27		0.59			84	383
22	77	exitus letalis	226	5.24		9.94		11.5	303	1295
26	50	recovery	130	5.38				5.6	94	756
27	74	recovery	155.5	6.2		1.38		10.1	82	1297
32	79	exitus letalis	32.3	3.78		14.76		21.1	295	1122
36	48	recovery	69.4	5.88		0.39		9.0	71	1418
37	75	exitus letalis	205.9	8.0		1.99		478	424	1892
38	43	recovery	177.1	6.01		1.22		7.1	111	2324
51	73	recovery	40.7	7.35		0.7		5.3	87	237
53	98	recovery	245.5	5.1		0.73		3.2	82	439.0
54	48	exitus letalis	19.3	5.2		0.82		4.7	101	520
63	79	exitus letalis	229.8	6.07		2.19		13.1	138	604
65	66	exitus letalis	140	4.93		3.56		17.3	194	776
66	72	recovery	124.6	7.5		1.6		5.1	115	492
77	82	recovery	170	6.27		7.94		13.7	153	1266
80	49	recovery	60.8	5.38		1.03		4.4	102	259
84	64	recovery	104.9	6.17		0.50		5.4	63	405
89	62	exitus letalis	71.6	3.57		1.25		20.6	478	553
91	62	exitus letalis	183.2	5.31		0.86		24.7	236	597

## Data Availability

Data are available upon reasonable request to the Corresponding Author.

## References

[1] Latorre D. (2022). Autoimmunity and SARS-CoV-2 infection: Unraveling the link in neurological disorders. Eur. J. Immunol..

[2] Cavalli E., Petralia M.C., Basile M.S., Bramanti A., Bramanti P., Nicoletti F., Spandidos D.A., Shoenfeld Y., Fagone P. (2020). Transcriptomic analysis of COVID-19 lungs and bronchoalveolar lavage fluid samples reveals predominant B cell activation responses to infection. Int. J. Mol. Med..

[3] Dharra R., Sharma A.K., Datta S. (2023). Emerging aspects of cytokine storm in COVID-19: The role of proinflammatory cytokines and therapeutic prospects. Cytokine.

[4] Maison D.P., Deng Y., Gerschenson M. (2023). SARS-CoV-2 and the host-immune response. Front. Immunol..

[5] Du T., Gao C., Lu S., Liu Q., Yu W., Li W., Sun Y.Q., Tang C., Wang J., Gao J. Differential Transcriptomic Landscapes of SARS-CoV-2 Variants in Multiple Organs from Infected Rhesus Macaques. Genom. Proteom. Bioinform..

[6] Xu J., Li X.-X., Yuan N., Li C., Yang J.-G., Cheng L.-M., Lu Z.-X., Hou H.-Y., Zhang B., Hu H. (2023). T cell receptor β repertoires in patients with COVID-19 reveal disease severity signatures. Front. Immunol..

[7] Taylor L. (2022). COVID-19: True global death toll from pandemic is almost 15 million, says WHO. BMJ.

[8] Martonik D., Parfieniuk-Kowerda A., Starosz A., Grubczak K., Moniuszko M., Flisiak R. (2023). Effect of antiviral and immunomodulatory treatment on a cytokine profile in patients with COVID-19. Front. Immunol..

[9] Günther S., Fagone P., Jalce G., Atanasov A.G., Guignabert C., Nicoletti F. (2019). Role of MIF and D-DT in immune-inflammatory, autoimmune, and chronic respiratory diseases: From pathogenic factors to therapeutic targets. Drug Discov. Today.

[10] Toldi J., Kelava L., Marton S., Muhl D., Kustan P., Feher Z., Maar K., Garai J., Pakai E., Garami A. (2023). Distinct patterns of serum and urine macrophage migration inhibitory factor kinetics predict death in sepsis: A prospective, observational clinical study. Sci. Rep..

[11] Ferreira P.T.M., Oliveira-Scussel A.C.M., Sousa R.A.P., Gomes B.Q., Félix J.E., Silva R.J., Millian I.B., Assunção T.S.F., Teixeira S.C., Gomes M.d.L.M. (2023). Macrophage Migration Inhibitory Factor contributes to drive phenotypic and functional macrophages activation in response to Toxoplasma gondii infection. Immunobiology.

[12] Huang G., Ma L., Shen L., Lei Y., Guo L., Deng Y., Ding Y. (2022). MIF/SCL3A2 depletion inhibits the proliferation and metastasis of colorectal cancer cells via the AKT/GSK-3β pathway and cell iron death. J. Cell. Mol. Med..

[13] Garcia-Gerique L., García M., Garrido-Garcia A., Gómez-González S., Torrebadell M., Prada E., Pascual-Pasto G., Muñoz O., Perez-Jaume S., Lemos I. (2022). MIF/CXCR4 signaling axis contributes to survival, invasion, and drug resistance of metastatic neuroblastoma cells in the bone marrow microenvironment. BMC Cancer.

[14] Zan C., Yang B., Brandhofer M., El Bounkari O., Bernhagen J. (2022). D-dopachrome tautomerase in cardiovascular and inflammatory diseases—A new kid on the block or just another MIF?. FASEB J..

[15] Barthelmess R.M., Stijlemans B., Van Ginderachter J.A. (2023). Hallmarks of Cancer Affected by the MIF Cytokine Family. Cancers.

[16] Sun H., Cheng R., Zhang D., Guo Y., Li F., Li Y., Li Y., Bai X., Mo J., Huang C. (2023). MIF promotes cell invasion by the LRP1-uPAR interaction in pancreatic cancer cells. Front. Oncol..

[17] Zhu G.-Q., Tang Z., Huang R., Qu W.-F., Fang Y., Yang R., Tao C.-Y., Gao J., Wu X.-L., Sun H.-X. (2023). CD36+ cancer-associated fibroblasts provide immunosuppressive microenvironment for hepatocellular carcinoma via secretion of macrophage migration inhibitory factor. Cell Discov..

[18] Fang T., Liu L., Song D., Huang D. (2023). The role of MIF in periodontitis: A potential pathogenic driver, biomarker, and therapeutic target. Oral Dis..

[19] Huth S., Huth L., Heise R., Marquardt Y., Lopopolo L., Piecychna M., Boor P., Fingerle-Rowson G., Kapurniotu A., Yazdi A.S. (2023). Macrophage migration inhibitory factor (MIF) and its homolog D-dopachrome tautomerase (D-DT) are significant promotors of UVB-but not chemically induced non-melanoma skin cancer. Sci. Rep..

[20] Du X., Li R., Song S., Ma L., Xue H. (2020). The Role of MIF-173G/C Gene Polymorphism in the Susceptibility of Autoimmune Diseases. Mediat. Inflamm..

[21] Gupta P., Joshi N., Uprety S., Dogra S., De D., Handa S., Minz R.W., Singh S., Chhabra S. (2021). Association of MIF gene polymorphisms with pemphigus vulgaris: A case-control study with comprehensive review of the literature. Int. J. Clin. Exp. Pathol..

[22] Thiele M., Donnelly S.C., Mitchell R.A. (2022). OxMIF: A druggable isoform of macrophage migration inhibitory factor in cancer and inflammatory diseases. J. Immunother. Cancer.

[23] Nguyen M.T., Beck J., Lue H., Fünfzig H., Kleemann R., Koolwijk P., Kapurniotu A., Bernhagen J. (2003). A 16-Residue Peptide Fragment of Macrophage Migration Inhibitory Factor, MIF-(50–65), Exhibits Redox Activity and Has MIF-like Biological Functions. J. Biol. Chem..

[24] Harris J., VanPatten S., Deen N.S., Al-Abed Y., Morand E.F. (2019). Rediscovering MIF: New Tricks for an Old Cytokine. Trends Immunol..

[25] Kang I., Bucala R. (2019). The immunobiology of MIF: Function, genetics and prospects for precision medicine. Nat. Rev. Rheumatol..

[26] Merk M., Mitchell R.A., Endres S., Bucala R. (2012). D-dopachrome tautomerase (D-DT or MIF-2): Doubling the MIF cytokine family. Cytokine.

[27] Tilstam P.V., Pantouris G., Corman M., Andreoli M., Mahboubi K., Davis G., Du X., Leng L., Lolis E., Bucala R. (2019). A selective small-molecule inhibitor of macrophage migration inhibitory factor-2 (MIF-2), a MIF cytokine superfamily member, inhibits MIF-2 biological activity. J. Biol. Chem..

[28] Aksakal A., Kerget B., Kerget F., Aşkın S. (2021). Evaluation of the relationship between macrophage migration inhibitory factor level and clinical course in patients with COVID-19 pneumonia. J. Med. Virol..

[29] Bleilevens C., Soppert J., Hoffmann A., Breuer T., Bernhagen J., Martin L., Stiehler L., Marx G., Dreher M., Stoppe C. (2021). Macrophage Migration Inhibitory Factor (MIF) Plasma Concentration in Critically Ill COVID-19 Patients: A Prospective Observational Study. Diagnostics.

[30] Dheir H., Yaylaci S., Sipahi S., Genc A.C., Cekic D., Tuncer F.B., Cokluk E., Kocayigit H., Genc A.B., Salihi S. (2021). Does Macrophage Migration Inhibitory Factor predict the prognosis of COVID-19 disease?. J. Infect. Dev. Ctries..

[31] Del Valle D.M., Kim-Schulze S., Huang H.-H., Beckmann N.D., Nirenberg S., Wang B., Lavin Y., Swartz T.H., Madduri D., Stock A. (2020). An inflammatory cytokine signature predicts COVID-19 severity and survival. Nat. Med..

[32] Langnau C., Janing H., Kocaman H., Gekeler S., Günter M., Petersen-Uribe Á., Jaeger P., Koch B., Kreisselmeier K.-P., Castor T. (2023). Recovery of systemic hyperinflammation in patients with severe SARS-CoV-2 infection. Biomarkers.

[33] Arunachalam P.S., Wimmers F., Mok C.K.P., Perera R.A.P.M., Scott M., Hagan T., Sigal N., Feng Y., Bristow L., Tsang O.T.-Y. (2020). Systems biological assessment of immunity to mild versus severe COVID-19 infection in humans. Science.

[34] Al Mahi N., Najafabadi M.F., Pilarczyk M., Kouril M., Medvedovic M. (2019). GREIN: An Interactive Web Platform for Re-analyzing GEO RNA-seq Data. Sci. Rep..

[35] Shin J.J., Fan W., Par-Young J., Piecychna M., Leng L., Israni-Winger K., Qing H., Gu J., Zhao H., Schulz W.L. (2023). MIF is a common genetic determinant of COVID-19 symptomatic infection and severity. QJM.

[36] Rajasekaran D., Zierow S., Syed M., Bucala R., Bhandari V., Lolis E.J. (2014). Targeting distinct tautomerase sites of D-DT and MIF with a single molecule for inhibition of neutrophil lung recruitment. FASEB J..

[37] Keene J.D. (2001). Ribonucleoprotein infrastructure regulating the flow of genetic information between the genome and the proteome. Proc. Natl. Acad. Sci. USA.

[38] Gibellini L., De Biasi S., Meschiari M., Gozzi L., Paolini A., Borella R., Mattioli M., Tartaro D.L., Fidanza L., Neroni A. (2022). Plasma Cytokine Atlas Reveals the Importance of TH2 Polarization and Interferons in Predicting COVID-19 Severity and Survival. Front. Immunol..

[39] Pons M.J., Ymaña B., Mayanga-Herrera A., Sáenz Y., Alvarez-Erviti L., Tapia-Rojas S., Gamarra R., Blanco A.B., Moncunill G., Ugarte-Gil M.F. (2021). Cytokine Profiles Associated With Worse Prognosis in a Hospitalized Peruvian COVID-19 Cohort. Front. Immunol..

[40] Montazersaheb S., Khatibi S.M.H., Hejazi M.S., Tarhriz V., Farjami A., Sorbeni F.G., Farahzadi R., Ghasemnejad T. (2022). COVID-19 infection: An overview on cytokine storm and related interventions. Virol. J..

[41] Donlan A.N., Sutherland T.E., Marie C., Preissner S., Bradley B.T., Carpenter R.M., Sturek J.M., Ma J.Z., Moreau G.B., Donowitz J.R. (2021). IL-13 is a driver of COVID-19 severity. J. Clin. Investig..

[42] Nicoletti F., Mancuso G., Cusumano V., Di Marco R., Zaccone P., Bendtzen K., Teti G. (1997). Prevention of endotoxin-induced lethality in neonatal mice by interleukin-13. Eur. J. Immunol..

[43] Baumhofer J.M., Beinhauer B.G., Wang J.E., Brandmeier H., Geissler K., Losert U., Philip R., Aversa G., Rogy M.A. (1998). Gene Transfer with IL-4 and IL-13 Improves Survival in Lethal Endotoxemia in the Mouse and Ameliorates Peritoneal Macrophages Immune Competence. Eur. J. Immunol..

[44] Muchamuel T., Menon S., Pisacane P., Howard M.C., A Cockayne D. (1997). IL-13 protects mice from lipopolysaccharide-induced lethal endotoxemia: Correlation with down-modulation of TNF-alpha, IFN-gamma, and IL-12 production. J. Immunol..

[45] Szabo S., Zayachkivska O., Hussain A., Muller V. (2023). What is really ‘Long COVID’?. Inflammopharmacology.

[46] Cavalli E., Mazzon E., Basile M.S., Mangano K., Di Marco R., Bramanti P., Nicoletti F., Fagone P., Petralia M.C. (2019). Upregulated Expression of Macrophage Migration Inhibitory Factor, Its Analogue D-Dopachrome Tautomerase, and the CD44 Receptor in Peripheral CD4 T Cells from Clinically Isolated Syndrome Patients with Rapid Conversion to Clinical Defined Multiple Sclerosis. Medicina.

[47] Fagone P., Mazzon E., Cavalli E., Bramanti A., Petralia M.C., Mangano K., Al-Abed Y., Bramati P., Nicoletti F. (2018). Contribution of the macrophage migration inhibitory factor superfamily of cytokines in the pathogenesis of preclinical and human multiple sclerosis: In silico and in vivo evidences. J. Neuroimmunol..

[48] Benedek G., Meza-Romero R., Jordan K., Zhang Y., Nguyen H., Kent G., Li J., Siu E., Frazer J., Piecychna M. (2017). MIF and D-DT are potential disease severity modifiers in male MS subjects. Proc. Natl. Acad. Sci. USA.

[49] Han Z., Qu J., Zhao J., Zou X. (2018). Genetic Variant rs755622 Regulates Expression of the Multiple Sclerosis Severity Modifier D-Dopachrome Tautomerase in a Sex-Specific Way. BioMed Res. Int..

[50] Nicoletti F., Créange A., Orlikowski D., Bolgert F., Mangano K., Metz C., Di Marco R., Al Abed Y. (2005). Macrophage migration inhibitory factor (MIF) seems crucially involved in Guillain–Barré syndrome and experimental allergic neuritis. J. Neuroimmunol..

[51] Laudanski K., Hajj J., Restrepo M., Siddiq K., Okeke T., Rader D.J. (2021). Dynamic Changes in Central and Peripheral Neuro-Injury vs. Neuroprotective Serum Markers in COVID-19 Are Modulated by Different Types of Anti-Viral Treatments but Do Not Affect the Incidence of Late and Early Strokes. Biomedicines.

